# Genomic selection analysis of morphological and adaptation traits in Chinese indigenous dog breeds

**DOI:** 10.3389/fvets.2023.1237780

**Published:** 2023-09-15

**Authors:** Yangfeng Li, Min Huang, Zhenjie Wang, Xueyuan Liu, Shan He, Tao Wang, Baicheng Ma, Jianyun Liu, Xingnuan Li, Jianjun Xiong, Jinlian Hua, Junhua Ye, Anmin Lei, Qianyong Yang

**Affiliations:** ^1^College of Veterinary Medicine, Northwest A&F University, Xianyang, China; ^2^College of Animal Science and Technology, College of Veterinary Medicine, Zhejiang A&F University, Hangzhou, China; ^3^Jiangxi Provincial Key Laboratory of Systems Biomedicine, Jiujiang University, Jiujiang, China; ^4^School of Forensic Medicine, Southern Medical University, Guangzhou, China; ^5^Jiujiang Key Laboratory of Rare Disease Research, Jiujiang University, Jiujiang, China; ^6^Medical College of Nanchang Institute of Technology, Nanchang Institute of Technology, Nanchang, China

**Keywords:** Chinese indigenous dogs, selection sweep, *F*_ROH_, *F*_ST_, morphological traits, adaptation traits

## Abstract

The significant morphological differences and abundant germplasm resources of Chinese indigenous dog breeds can be attributed to the diverse geographical environment, including plateaus, mountains, and a long history of raising dogs. The combination of both natural and artificial selection during the past several thousand years has led to hundreds of dog breeds with distinct morphological traits and environmental adaptations. China is one of the earliest countries to domesticate dogs and there are more than 50 ancient indigenous dog breeds. In this study, the run of homozygosity (ROH) and proportion of the autosomal genome covered by ROHs (*F*_ROH_) were calculated for 10 dog breeds that are the most representative Chinese indigenous dogs based on 170K SNP microarray. The results of *F*_ROH_ showed that the Chuandong hound dogs (HCSSC) have the highest level of inbreeding among the tested breeds. The inbreeding in HCSSC occurred more recently than the Liangshan dogs (SCLSQ) dogs because of more numbers of long ROHs in HCSSC dogs, and the former also have higher inbreeding degree. In addition, there are significant differences in the inbreeding degree among different subpopulations of the same breed, such as the Thin dogs from Shaanxi and Shandong province. To explore genome-wide selection signatures among different breeds, including coat color, ear shape, and altitude adaptability, we performed genome selection analyses of *F*_ST_ and cross population extended haplotype homozygosity (XP-EHH). For the coat color, the *F*_ST_ analysis between Xiasi dogs (XSGZ) and HCSSC dogs was performed and identified multiple genes involved in coat color, hair follicle, and bone development, including *MC1R*, *KITLG*, *SOX5*, *RSPO2*, and *TBX15*. For the plateau adaptability, we performed *F*_ST_ and XP-EHH analyses between dogs from Tibet (Tibetan Mastiffs and Nyingchi dogs) and plain regions (Guangxi Biwei dogs GXBWQ and Guandong Sharpei dogs). The results showed the *EPAS1* gene in dogs from Tibet undergo strong selection. Multiple genes identified for selection signals based on different usage of dogs. Furthermore, the results of ear shape analyses showed that *MSRB3* was likely to be the main gene causing the drop ear of domestic dogs. Our study provides new insights into further understanding of Chinese indigenous dogs.

## 1. Introduction

The dogs were the first domesticated animal in human history (1) and originated from gray wolf (2) with strong morphological selection during domesticating progress. The researchers found that the dog originated from Southern East Asia (3, 4), the Middle East (5), Europe (6) and Central Asia (7), which means the origin of modern dog breeds is controversial until now. The period of significant morphological divergence from the gray wolf likely occurred about 10,000 to 15,000 years ago (8). However, a much shorter time of morphological divergence occurred in modern dog breeds. During the past several hundred years, humans have bred numbers of dog breeds through inbreeding, which led to decline of the genetic diversity in these dog breeds (9). The current purebred dogs have been bred for conformation based on the adherence to “breed standards” specified by international associations, such as the American Kennel Club (AKC) and The Federation Cynologique Internationale (FCI). Globally, more than 400 dog breeds were registered in these associations (10). The history of domesticated dog in China was over 10,000 years (11–15) and more than 50 breeds with stable genetic characteristic were retained after long time artificial selection, such as ancient Guangdong Shapi dogs, Thin dogs, and Tibetan Mastiff. Chinese indigenous dogs have markedly difference from their ancestor gray wolves, especially their morphological characteristics (16). Exploration of genes related to morphology characteristics and environmental suitability will be beneficial to the stable inheritance of purebred dog and breed new dogs. For example, Cadieu et al. (17) performed genome-wide association studies of 80 domestic breeds to identify genes associated with canine fur phenotypes and identified distinct mutations in *RSPO2*, *FGF5*, and *KRT71* that collectively account for most coat phenotypes in purebred dogs in the United States. Besides, the *IGF1* gene was found determined the body size of dogs (18), and several genes are found to play important roles in coat color phenotype of the canine (19).

Although China has abundant germplasm resources of dog breeds, there has been insufficient attention on these Chinese indigenous dog breeds, resulting in limited researches about their morphological characteristics, including coat color, body type, and ear type. According to the 2021 Veterinary Medicine White Paper (Industry Research Report), China has more than 52.22 million dogs and the majority of dog breeds are western breeds (more than 92%) which means that Chinese do not pay much attention to native dogs and Chinese indigenous dogs need to be protected urgently. The traditional breeding practices of Chinese indigenous dog breeds are still retained in some rural areas, which maintains the original pedigree of these dog breeds due to especial geography and culture. In this study, we performed several analyses to explore the inbreeding degree of 10 Chinese ancient dog breeds and their special characteristic using 170K high-density SNP microarrays, such as coat color, ear shape, and high-altitude adaptation.

## 2. Materials and methods

### 2.1. Data

The 170K high-density SNP microarrays data used in this study was previously published (20), and the microarrays was designed based on the reference of CanFam version 3.1. A total of 130 individuals and 144,481 single nucleotide polymorphism sites (SNPs) were retain by PLINK v1.9 with minimum allele frequency (MAF) >0.01. The 130 individuals representing 10 breeds and 14 populations (Supplementary Table S1). There are two populations of the Thin dogs, including population from Shaanxi and Shandong province. The Tibetan Mastiff are divided into four populations according to their coat color.

### 2.2. Estimation of the inbreeding degree

Runs of homozygosity (ROHs) were calculated using the command --homozyg --homozyg-snp 20 --homozyg-kb 500 by PLINK v1.9 software, where --homozyg-snp indicates a minimum number of SNPs of 20 and --homozyg-kb indicates a minimum homozygous fragment length of 500 kb. The numbers of fragment length <1 Mb, 1–5 Mb, 5–10 Mb, and >10 Mb were counted, and the proportion of the autosomal genome covered by ROHs (*F*_ROH_) of each individual was calculated using the formula Total_ROH_/*L*_genome_, where Total_ROH_ is the sum of the ROH lengths of each population, *L*_genome_ is the size of the dog’s autosome, and the average value of *F*_ROH_ of all individuals is the *F*_ROH_ of the population.

### 2.3. Genomic selective sweep analysis

The Chinese indigenous dog breeds used in this study are ancient breeds with significant phenotypic differences. These populations were divided into several categories based on phenotypes, places of origin, and usage. We then performed *F*_ST_ and XP-EHH analyses using VCFtools v1.6 software, with the top 0.5% SNP loci as outliers. The categories were divided as follows: (1) coat color was divided into white and black-yellow groups, the Guizhou Xiasi dogs (XSGZ) were designated as the white group and the HCSSC designated as the black-yellow group; (2) the Tibetan mastiff (including four populations of TCG, TCHQ, TCW, and TCY, Supplementary Table S1) and Nyingchi dogs (TCLZ) were designated as the high-altitude group, the Guangxi Biwei dogs (GXBWQ) and Guandong Sharpei dogs (SPGD) were designated as the tropical low altitude group; (3) according to the usage, the SPGD dogs were designated as the bulldog group, and the GXBWQ, XSGZ, and Liangshan (SCLSQ) and Sichuan Qingchuan (SCQCQ) dogs (SC) were designated as the guard group; (4) according to the ear type, the GXBWQ, HCSSC and XSGZ dogs were designated as erect ear group, the SCLSQ and SCQCQ dogs were designated as drop ear group.

### 2.4. Gene functional enrichment analysis

The outlier SNPs identified from genomic selective sweep analyses were used to compare with the annotation to recognize selective genes. Functional enrichment analyses of these selective genes were performed using the Metascape website^1^ and clusterProfiler package in R language.

## 3. Results

### 3.1. ROH-based estimation of inbreeding degree in Chinese indigenous dog breeds

We found that there was significant difference of the number of ROHs in different Chinese indigenous dog breeds (Table 1), which may be caused by different number of individuals in different dog breeds. Actually, the ROH fragment lengths were mainly concentrated in 1–5 Mb in all breeds. The populations of GXBWQ, SCLSQ, SCQCQ, SPGD, TCLZ, Shandong Thin dogs (ThCSD), and Shaanxi Thin dogs (ThCSX) have similar numbers of individuals, however, the numbers of ROH are significant differences in these populations. Here, the ThCSX have the highest number of ROHs (*n* = 1809) and the ThCSD have the lowest number (*n* = 482). Interestingly, both ThCSD and ThCSX were the Chinese Thin dogs, but the number of ROHs were obvious difference between the two populations (482 vs. 1809), which suggested that the inbreeding degree of the ThCSX dogs was much higher than that of ThCSD dogs. The *F*_ROH_ results also reflect the inbreeding condition and show that GXBWQ, XSGZ, and some Tibetan Mastiff have lower inbreeding degree. The longer ROHs indicate that inbreeding has occurred in recent generations, while the shorter ROHs indicate that inbreeding was derived from more distant generations. Besides, a smaller number of generations indicates a lower likelihood that ROHs are interrupted by recombination (21). Both HCSSC and SCLSQ dogs have higher inbreeding coefficients (0.21 vs. 0.17), but the number of ROHs whose lengths are below 5 Mb in HCSSC dogs is lower than that of SCLSQ dogs, and the opposite is true for those above 5 Mb. This result suggested the time of inbreeding event occurred in the HCSSC dogs was earlier than the SCLSQ dogs. In addition, both recent and distant inbreeding events were found in the Chinese Thin dogs (Table 1). We used SNeP v1.1 software (22) to estimate the recent effective population size (Ne) of each breed, and the results showed that the XSGZ dogs have the largest Ne while the SPGD dogs have opposite Ne under similar numbers of individuals (Table 1).

**Table 1:** Distribution of runs of homozygosity and recently effective population size of each population.

Abb.^a^	No.	0.5–1 Mb	1–5 Mb	5–10 Mb	>10 Mb	Total	*F*_ROH_	Ne
ColKZ	5	56	95	8	28	187	0.10	15
GXBWQ	12	263	306	12	32	613	0.07	56
HCSSC	12	284	683	141	139	1,247	0.21	41
SCLSQ	12	478	881	78	79	1,516	0.17	40
SCQCQ	12	248	547	74	67	936	0.12	53
SPGD	10	150	259	73	92	574	0.16	24
TCG	5	120	163	12	10	305	0.08	20
TCHQ	7	123	185	7	13	328	0.07	24
TCLZ	12	289	529	42	50	910	0.11	53
TCW	5	57	95	4	12	168	0.06	20
TCY	4	70	137	17	43	267	0.18	13
ThCSD	10	122	256	39	65	482	0.11	38
ThCSX	12	349	1,046	257	157	1,809	0.26	36
XSGZ	12	238	337	9	32	616	0.08	62

a See Supplementary Table S1 for group abbreviations and breeds.

### 3.2. Selective sweep analysis of hair type and body size in domestic dogs

**Figure 1:**
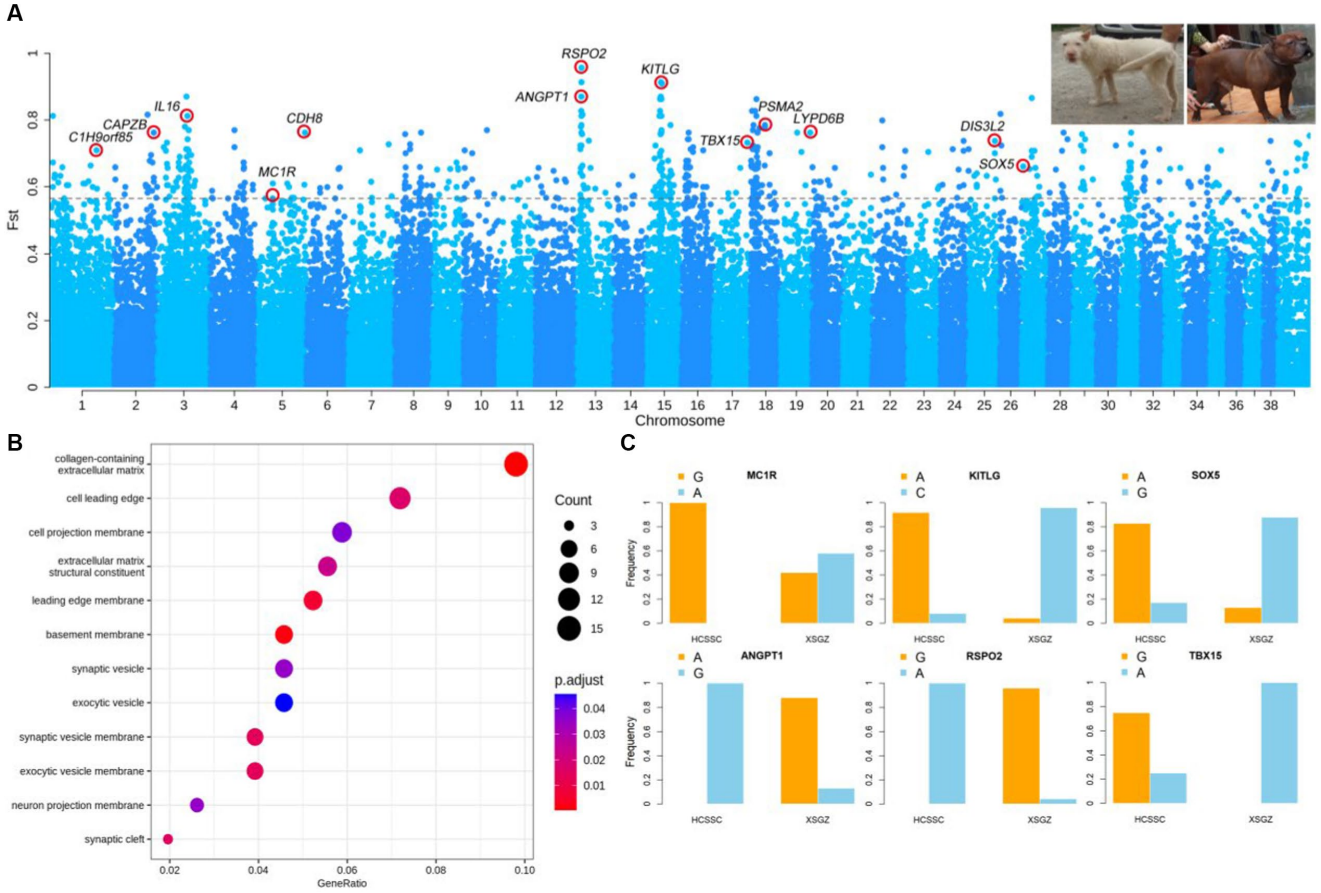
Results of dog coat color selection sweep analysis. **(A)** Manhattan map of *F*_ST_ analysis results comparing XSGZ and HCSSC groups. Each point represents an SNP, and the gray dashed line indicates the top 0.5% threshold. The top right image shows the XSGZ group on the left and the HCSSC group on the right. **(B)** Gene functional enrichment analysis. Each circle represents a GO term. **(C)** Allele frequencies of the *KITLG*, *MC1R*, *SOX5*, *TBX15*, and *RSPO2* genes in the XSGZ and HCSSC groups. XSGZ, Guizhou Xiasi dog; HCSSC, Chuandong hound dog.

The *F*_ST_ analysis was performed between XSGZ (white coat color) and HCSSC (black-yellow coat color) dogs and the value of the top 0.5% of SNPs (*F*_ST top 0.5%_ = 0.56) was set as threshold. We identified 689 SNPs above the threshold and annotated 165 genes (Figure 1A and Supplementary Table S2). Functional enrichment analysis revealed that these genes are mostly involved in several biological processes. Several genes determined to be related to coat color, hair follicle, and bone development were identified, including *MC1R*, *KITLG*, *SOX5*, *RSPO2*, and *TBX15* (Figure 1B). The *MC1R* has been reported to be a switch gene in pigmentation (23), *SOX5* affects *MITF-M* expression and melanogenesis in mouse skin melanocytes (24), and the *KITLG* gene has been reported to be associated with coat color in dogs (25). The genotype frequencies of the SNPs associated with genes of *MC1R*, *KITLG*, *SOX5*, *RSPO2*, *ANGPT1*, and *TBX15* were analyzed, and significant differences were found between the XSGZ and HCSSC dogs (Figure 1C). Functional annotation of mutations show that these mutations related to these genes are intronic variants using VEP tool. Among them, the genotype of the *MC1R* gene was homozygous G allele in HCSSC dogs, and the XSGZ dogs have heterozygous A/G alleles, whereas *KITLG* and *SOX5* genes showed opposite allele distributions in the two dog breeds. Reports have shown that the *ANGPT1* gene can promote vascular remodeling, maturation, and sprouting and branching during angiogenesis (26), which is consistent with the fact that hounds are good at running and have strong cardiopulmonary function. The genotype distribution of *ANGPT1* showed almost complete difference between the two dog breeds. Actually, XSGZ dogs have a long and soft hair, while HCSSC dogs have short and hard hair (Figure 1A). Here, we identified *RSPO2* gene which has been reported to be involved in softness and length of hair (27), and the allele frequencies of the SNPs related to the *RSPO2* gene was almost completely different in the two dog breeds. Genotype frequency analysis showed that the G allele was nearly homozygous in the XSGZ dogs and that the A allele genotype was present in all other breeds (Supplementary Table S3). Actually, the XSGZ dogs have characteristic of two hair layers. Thus, we hypothesized that *RSPO2* may affect hair growth of the XSGZ breed, especially their length and softness. The HCSSC dogs are well-known Chinese indigenous hunting dogs with sturdier body than that of XSGZ dogs. We identified the *TBX15* gene that was reported to strong body (28). Besides, *TBX15* can help humans produce brown fat cells and it works with *WARS2* together to generate more energy against cold weather (29). We found the *TBX15* gene was strongly selected in large dogs and the allele frequency analysis showed that the frequency of allele A was higher that of G, such as Tibetan Mastiff and XSGZ dogs (Figure 1C and Supplementary Table S3).

### 3.3. Selective sweep analysis of high-altitude adaptation in domestic dogs

**Figure 2:**
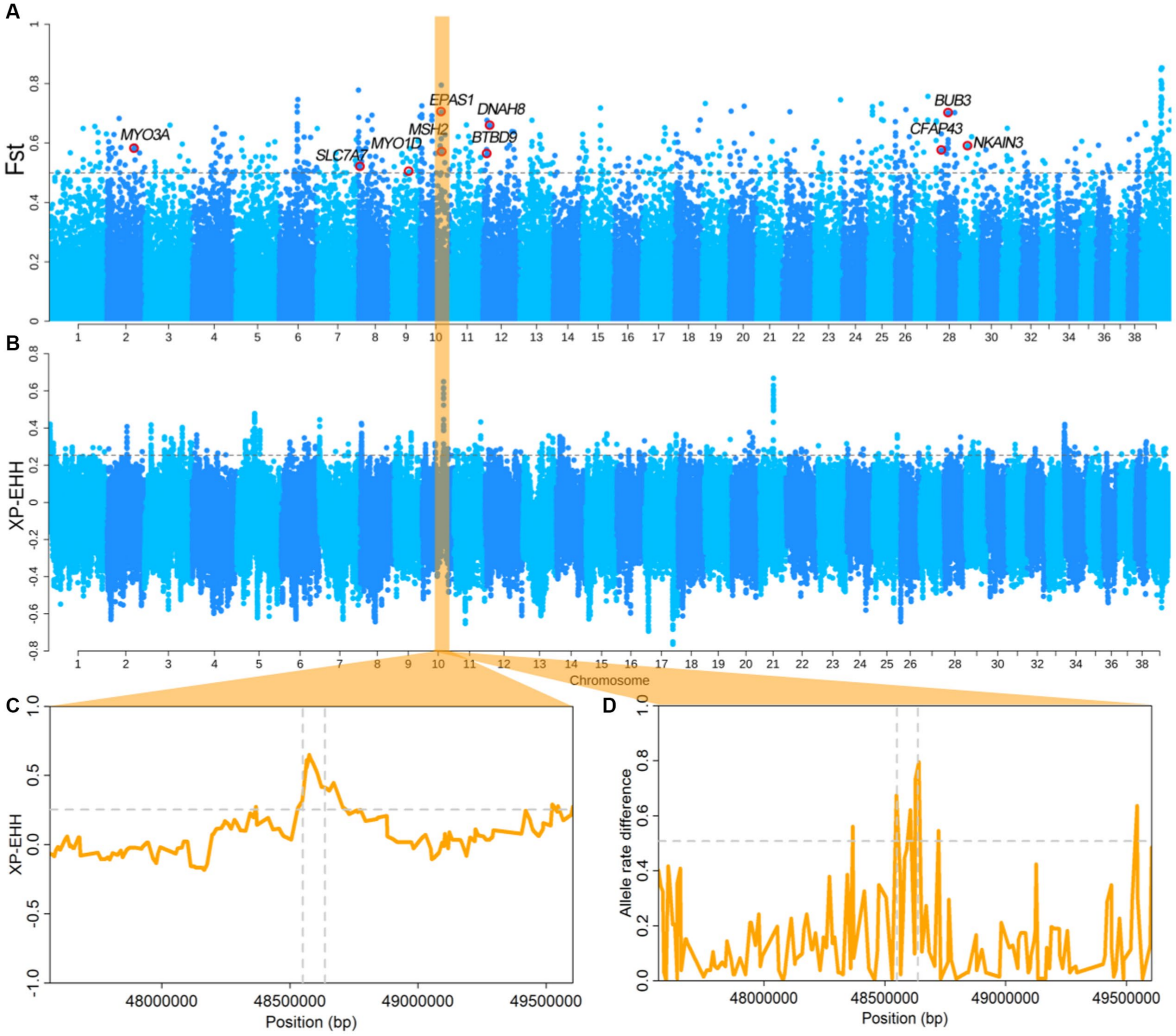
Results of dog type selection sweep analysis. **(A,B)** *F*_ST_ and EP-EHH between highland and tropical dogs. **(C,D)** XP-EHH and allele differences of the *EPAS1* region. Dashed horizontal lines represent threshold lines at the genome level. The vertical dotted line shows the *EPAS1* gene region.

We divided the dogs from Tibet including TCG, TCHQ, TCW, TCY, and TCLZ (Supplementary Table S1) into high-altitude group, while SPGD and GXBWQ from Guangdong and Guangxi province as low altitude group. The *F*_ST_ and XP-EHH analyses were performed between the two groups. The values top 0.5% of SNPs were used as the threshold (*F*_ST top 0.5%_ = 0.5, XP-EHH_top 0.5%_ = 0.25), and total of ten overlapping genes were identified using the two methods (Figures 2A,B). Among which, the *EPAS1* was recognized and numerous reports showed that the *EPAS1* is an important gene for the evolution of high-altitude adaptation in animals, including human, pigs, cattle, and so on. The XP-EHH values and allele frequency difference of the *EPAS1* gene region were significantly higher than the threshold (Figures 2C,D), indicating that *EPAS1* have been strongly selected in the high-altitude dogs.

### 3.4. Genomic selection analysis of different usage in domestic dog breeds

**Figure 3:**
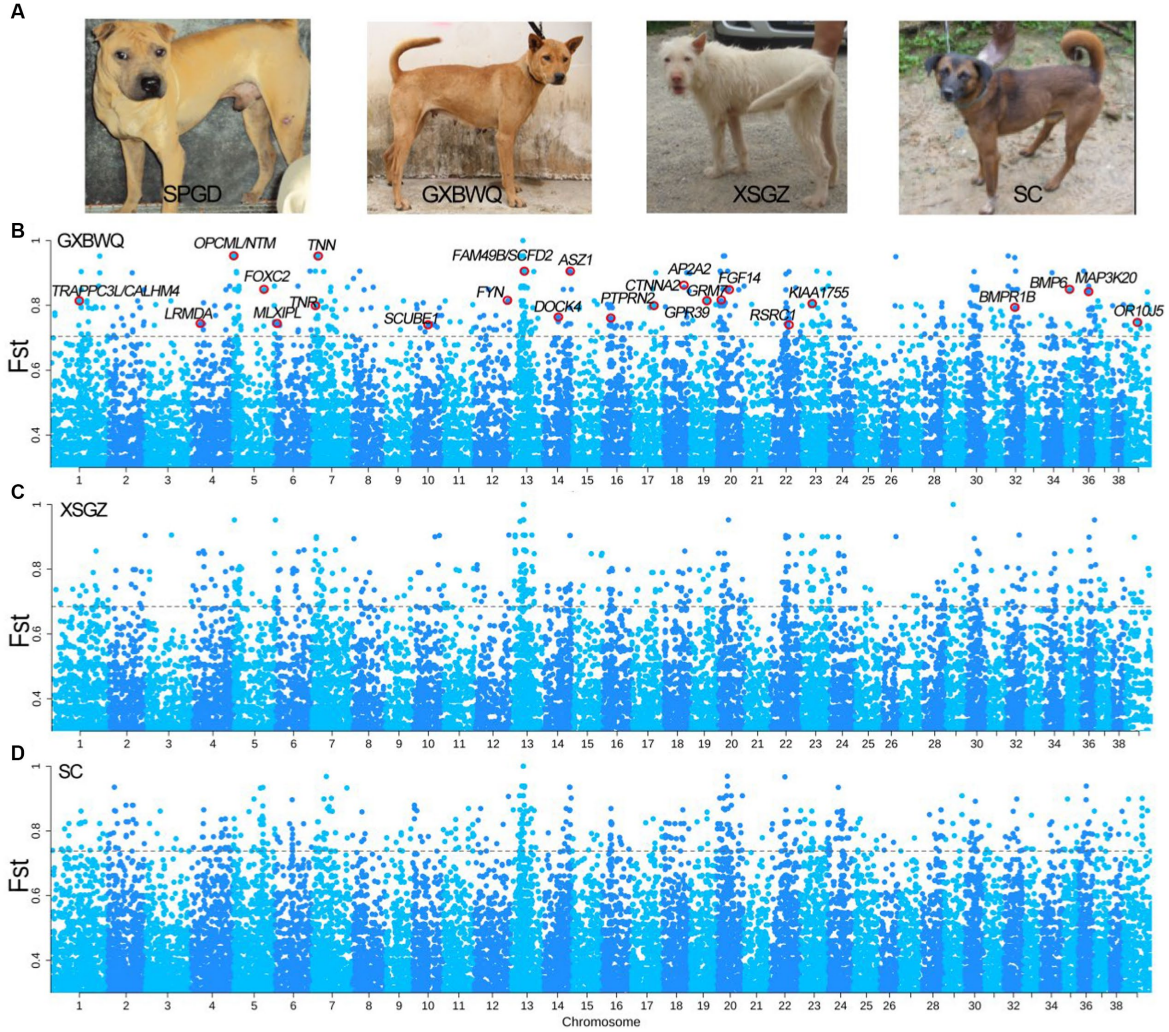
Results of dog type selection sweep analysis. **(A)** Photos of different dog breeds; **(B–D)** *F*_ST_ comparison among GXBWQ, XSGZ, and SC dogs, respectively. GXBWQ, Guangxi Biwei dogs; XSGZ, Guizhou Xiasi dogs; SC, Sichuan Qingchuan dogs.

We, respectively, performed *F*_ST_ analysis between SC dogs and other dog breeds of SPGD and GXBWQ, and XSGZ (Figure 3A), and 672, 662, and 680 SNPs (*F*_ST top 0.5%_ = 0.70, 0.68, and 0.74 respectively) were identified. Total of 172, 199, and 208 annotated genes were identified from the three groups (Figures 3B–D), of which, 48 genes were overlapping (Supplementary Table S4). Allele frequency analysis of these 48 genes (Supplementary Table S5) showed that there was significant difference between SPGD and the other dogs. Functional enrichment analysis of these 48 genes (Supplementary Figure S1) showed that these genes were primarily involved in progress of “neuron recognition” (GO:0008038), “regulation of chondrocyte differentiation” (GO:0032330), “growth” (GO:0040007), “regulation of synapse organization” (GO:0050807), and the genes involved in these processes mainly included *BMPR1B*, *GRM7*, *DOCK4*, *BMP6*, *NTM*, *TNN*, and *CNTN4*.

### 3.5. Genomic selection analysis of ear type in domestic dogs

**Figure 4:**
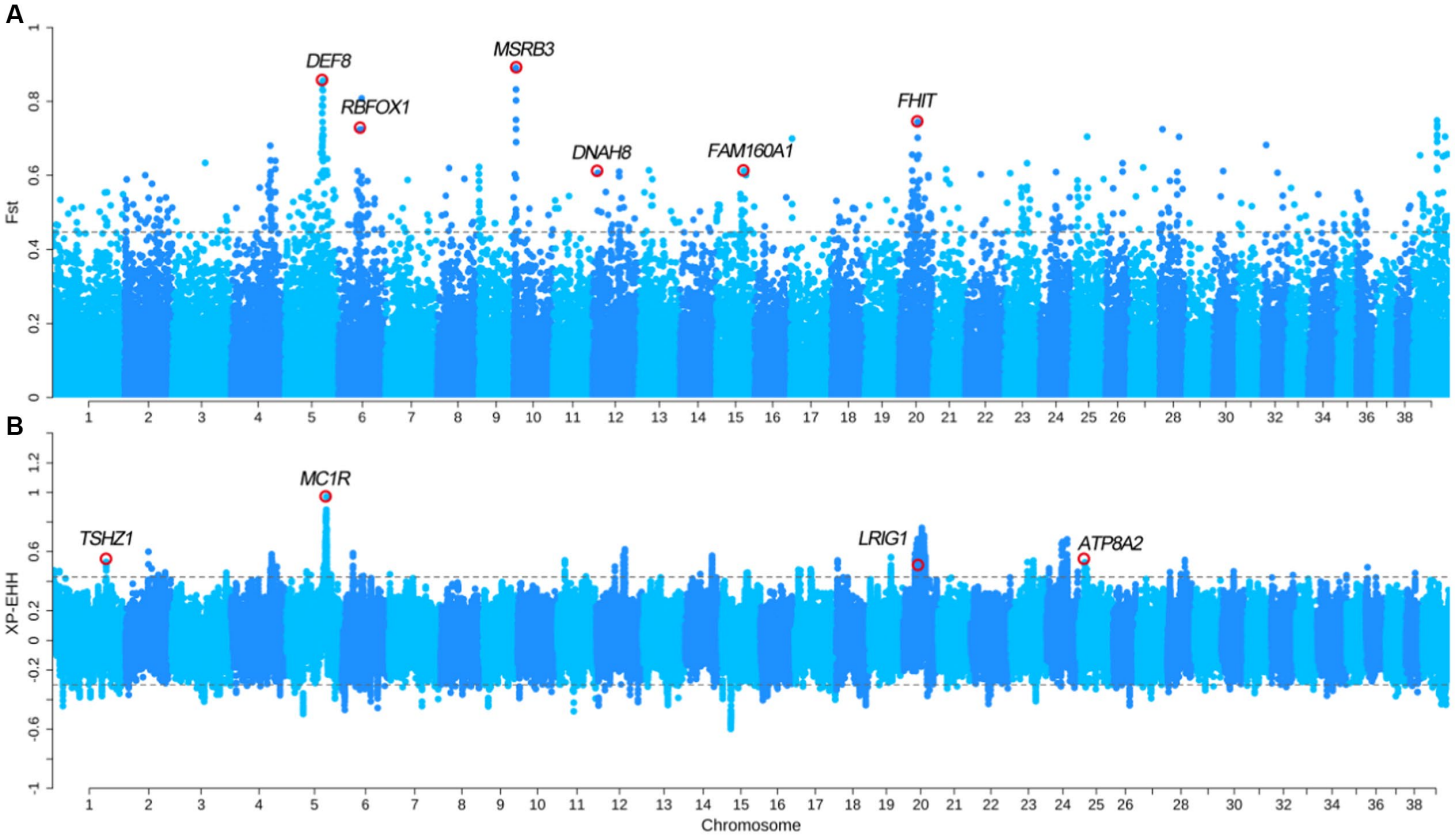
Selection sweep comparative analysis between erect ear group and drop ear group. **(A)** *F*_ST_ between erect ear and drop ear dogs; **(B)** XP-EHH between the two group dogs.

We divided the SCLSQ and SCQCQ dogs into the drop ear group, and GXBWQ, HCSSC, and XSGZ dogs into the erect ear group. The *F*_ST_ and XP-EHH analyses were performed (*F*_ST top 0.5%_ = 0.44 and XP-EHH_top 0.5%_ = 0.42) between the two groups. The *F*_ST_ analysis identified 183 genes (Figure 4A and Supplementary Table S6), and these genes primarily involved in some important biological processes (Supplementary Figure S3). In addition, *F*_ST_ analysis identified the *MSRB3* gene that is associated with ear shape and hearing development. Studies have shown that *MSRB3* is related to ear area, size, and type (30, 31). We hypothesis that the *MSRB3* is likely the major gene which caused drop ear in Chinese dogs since the shape and ear size between the drop and erect ear groups in our study is not significant. We further analyzed the genotype frequencies of the SNP related to *MSRB3* and found that the frequency of allele A in the GXBWQ, HCSSC, and XSGZ dogs with erect ears were almost 1 (Supplementary Table S7). Strangely, the SPGD dogs with small and drop ears had similarly allele frequency distribution with prick dogs. The XP-EHH analysis identified 101 genes (Figure 4B and Supplementary Table S8) and these genes mainly involved in several important biological processes (Supplementary Figure S4). Among these progresses, genes of *TSHZ1*, *LRIG1*, and *ATP8A2* were found to be involved in the “GO:0042471: ear morphogenesis” biological process. Interestingly, the XP-EHH also identified the *MC1R* gene that was associated with pigmentation. Besides, total of 24 overlapping genes were found from the two analyses, including *DEF8*, *RBFOX1*, *FHIT*, *TCF25*, and so on.

## 4. Discussion

### 4.1. The inbreeding status of Chinese indigenous dogs

China has a long history of domesticating dogs, and the germplasm resources of indigenous dogs are especially abundant. However, Chinese indigenous dogs are facing the problem of continuous loss of genetic resources under the impact of foreign famous dog breeds in the recent decades. In this study, we collected 130 samples from 10 ancient Chinese indigenous dog breeds and genotyped by 170K high-density SNP microarrays. We showed the inbreeding degree for each population based on ROH and found that significant differences in the inbreeding degree among populations. For example, the number of longer ROHs of the HCSSC dogs are significantly more than the GXBWQ dogs which means the inbreeding degree of former is much higher than that of the later. There are still significant differences between different populations of the same breed, such as the Chinese Thin dogs (ThCSD and ThCSX). The ThCSX dogs have the highest inbreeding degree among the 14 populations in this study, while the ThCSD dogs much lower than that of ThCSX dogs. The results implies that the genetic diversity of the ThCSD dogs is better, while ThCSX dogs is in dire need of conservation. Combined with the current status of pet dog breeding in China, these results suggest the urgency of the protection of Chinese indigenous dog germplasm resources. The results of recent effective population size analysis showed that some breeds have low Ne, especially SPGD, which suggested that SPGD needs to be protected urgently.

### 4.2. Genomic selection sweep analyses revealed several important traits in dogs

The breeding of varieties and speciation include the influences of both natural selection and artificial selection. Natural selection is a type of adaptive selection in the form of positive selection, for example, genetic mutations favoring adaptation to the environment appear in the population will be selected. With improved survival, a large change in the frequency of the locus corresponding to the mutation appears, generating population divergence. Dogs are the most important companions of human and play various roles in human life, including functions of companionship, guiding the blind, hunting, and search and rescue. Combined with human preferences for the appearance, dogs with various appearance characteristics have been bred during the past tens of thousands of years. Chinese indigenous dogs have been domesticated over a long history with strong artificial selection for different purpose of usage. The genetic diversity of Chinese indigenous dogs is influenced not only by the ecological environment of different region but also by the cultural environment and work purpose. The *ANGPT1* gene has been reported to promote vascular remodeling and maturation and facilitate sprouting and branching during angiogenesis (32) which is consistent with HCSSC hounds dogs being good runners and having strong cardiopulmonary function. Studies have shown significant differences in the expression of *RSPO2* in different hair types of the alpaca (27). We found the alleles SNP related to the *RSPO2* gene were almost opposite in the XSGZ and HCSSC dog breeds. In fact, the HCSSC dogs have short and hard hair, while the XSGZ dogs have the two hair layers, with one layer of long and soft and another of short and hard hair. This means the *RSPO2* gene is likely to be an important gene affecting the hardness and length of hair in XSGZ dogs. The GXBWQ, XSGZ, and SC dogs are excellent hunting dogs with eminent characteristics of climbing ability, strong fertility. Reports have shown that the *TBX15* in human was infiltrated from Neanderthals to resist cold climates. We found that the *TBX15* carried a selected mutation in the Tibetan Mastiff, which suggested that the mutation may play an important role in the cold resistance process of Tibetan Mastiffs. The *BMPR1B* gene encodes an important transmembrane receptor protein involved in the transforming growth factor β (TGF-β) pathway, which plays an important role in the regulation of osteogenic differentiation, cell spreading, and ovarian follicle development. The *BMPR1B* gene was reported affecting reproductive traits such as sheep (33). Genes including *GRM7*, *DOCK4*, and *CNTN4* affect brain and neural development (34–36). The selective effect is the main directional driver in this type of population differentiation, as observed in this study for the high-altitude adaptation genes including several genes, especially *EPAS1* gene which have been reported to be associated with natural selection for low hemoglobin concentrations (37). The pleiotropic adaptive effects of *EPAS1* was proposed to underlie the strong selective signaling in Tibetan species (38), including Tibetan cattle (39, 40), Tibetan swine (41), and Tibetan horses (42). In our study, we also found that *EPAS1* was under strong selection in Tibetan dogs. The relationship between *EPAS1* and lower hemoglobin content in Tibetan dogs needs to be further explored. Ear type is one of the important phenotypes of animals, especially domestic animals. Studies have shown that the *MSRB3* gene affects the ear area of pigs (31), while *MSRB3* gene may affect whether the ears droop in pigs (43). In this study, SCLSQ and SCQCQ were used as the drop ear group, and GXBWQ, HCSSC, and XSGZ were used as the upright ear group. The selection signals of the two groups identified the *MSRB3* gene. Considering that the ear areas of these two groups of dogs are not much different, we believe that the *MSRB3* is likely to be an important gene that affects whether the dog has erect ears. In summary, our research has laid the foundation for the exploration of Chinese indigenous dogs and their genetic resources protection.

## Data availability statement

Publicly available datasets were analyzed in this study. This data can be found here: https://datadryad.org/stash/landing/show?id=doi%3A10.5061%2Fdryad.76hdr7srv.

## Ethics statement

Ethical review and approval was not required for the study on animals in accordance with the local legislation and institutional requirements.

## Author contributions

QY and MH proposed the idea for the study. MH performed the bioinformatics analysis and wrote the paper. YL and MH directed the analyses and revised the article. ZW, XuL, SH, TW, BM, JL, XiL, JX, JH, JY, AL, and QY collected the samples and recorded the phenotypes. All authors contributed to the article and approved the submitted version.

## Funding

This work was supported by Doctoral Scientific Research Foundation of Jiujiang University (8741309).

## Conflict of interest

The authors declare that the research was conducted in the absence of any commercial or financial relationships that could be construed as a potential conflict of interest.

## Publisher’s note

All claims expressed in this article are solely those of the authors and do not necessarily represent those of their affiliated organizations, or those of the publisher, the editors and the reviewers. Any product that may be evaluated in this article, or claim that may be made by its manufacturer, is not guaranteed or endorsed by the publisher.

## Footnotes

^1^ http://metascape.org/
